# Catalysing transformational change through compound nature connectedness interventions

**DOI:** 10.1007/s13280-025-02328-0

**Published:** 2025-12-31

**Authors:** Matt Pritchard, Philip Tovey, Phoebe Tickell, Tom H. Oliver

**Affiliations:** 1 School of Biological Sciences, Health and Life Sciences Building, University of Reading, Whiteknights Campus, Reading, RG6 6AS UK; 2 Director of Nature-Centric Approaches, Accelerator for Systemic Risk Assessments (ASRA), London, UK; 3 Founder and Executive Director, Moral Imaginations, London, UK

**Keywords:** Compounding effects, Mindset change, Nature connectedness, Nature interventions, Polycrisis

## Abstract

Political, economic and technological interventions are struggling to address the polycrisis at the necessary scale and pace. Increasing nature connectedness can make a critical contribution: there is abundant evidence that higher levels of nature connectedness are not only good for health and happiness, but also correlate with a greater commitment to pro-environmental behaviours. The early progress in the scientific study of nature connectedness is impressive, but there are many types of interventions promoting nature connectedness, and evidence is fragmented across academic disciplines and siloed communities of practice. Here, we review a wide range of interventions and their effectiveness relative to each other. We appraise how interventions can be combined synergistically with the potential to catalyse shifts to pro-environmental mindsets through a concept of compounding effects. We conclude by discussing how our insights may inform the development of more holistic training and development approaches, as a critical element of sustainability transformations.

**Supplementary Information:**

The online version contains supplementary material available at 10.1007/s13280-025-02328-0.

## Introduction: Nature connectedness in an era of polycrisis

It is increasingly likely a mass extinction event on Earth is underway, driven by human activity (Diamond et al. 1989; Díaz et al. 2019; Smits and Finnegan 2019; Bergstrom et al. 2021; Wiens and Zelinka 2024; Wang et al. 2025). Environmental degradation—including population declines, species extinction, habitat fragmentation and loss of soil fertility—has been suggested as a key factor in socioecological collapse (Diamond 1994; Butzer 2012; Cumming and Peterson 2017; Rubiños and Anderies 2020; Beard et al. 2021), where collapse is defined as when “key actors, system components, and interactions [] disappear” (Cumming and Peterson 2017, p. 699). Withagen et al. (2025, p. 84) also suggest ecological collapse, as a concept, “…weaves a moral undercurrent, pointing toward a responsibility to change our lifestyle”. This has led some to call for society to transform its institutions, including policies and politics in order to enhance resilience (Brozovic 2023).

Evidence of this need for transformational change is now well established and consolidated in the Intergovernmental Platform on Biodiversity and Ecosystem Services (IPBES) Transformational Change Assessment (2024) ratified by 147 governments. It reports that “transformative change is urgent because there is a closing window of opportunity to avoid further biodiversity loss and prevent triggering the potentially irreversible decline and projected collapse of key ecosystem functions”, stating the need for fundamental system-wide shifts in views, structures and practices. It recognises “disconnection of people from nature” as one of the three underlying causes of biodiversity loss. To achieve transformational change, IPBES (2024) argues for shifting dominant societal views and values to prioritise and strengthen nature connectedness.

However, the landscape in which humans experience nature connectedness has changed—and is radically changing—from our ancestral landscape in which humans were closely affiliated with diverse lifeforms (Lumber et al. 2017), to our modern landscapes. The latter are now formed of densely urbanised artificiality of modern cities, changed demographies (Brewster et al. 2014), digital avatars and First Person View (FPV) drones (Lockhart et al. 2023), with the emergent conditions of the polycrisis “…shap[ing] everyday experience[s]…seriously reconfiguring…life routines…” as Golańska (2022, p. 125) documents. The Anthropocene, thus, presents deep complexity and challenges for strengthening nature connection.

In their “global synthesis of trends in human experience of nature”, Cazalis et al. (2023) evidence the changing composition of spaces humans inhabit as trending towards increased distance from natural areas, increased urbanisation and declining city forestry. Robert Pyle wrote of “the extinction of experience” of nature (Pyle 2011), yet Cazalis et al. (2023) failed to find supporting evidence, suggesting more nuance is needed in studying how interventions that might constitute experiences of nature are affecting nature connectedness. Soga and Gaston (2023, p. 136) support this in their global synthesis, revealing heterogeneous changes in nature connectedness, and that “the magnitude and direction of temporal changes in nature connection can vary among” affective, cognitive and experiential components. Recently, surveying 23 countries worldwide, the same authors found high variation in connection to nature, highlighting further “the multifaceted and complex nature of human connection to nature, emphasising the importance of considering multiple aspects to understand that connection” (Soga and Gaston 2025, p. 2). As the polycrisis deepens, taking a systemic view of nature connectedness interventions—across domains, through time and beyond borders (see Peirson et al. 2011)—will increasingly be needed to support policymakers’ (and others’) decision-making (Picanço Rodrigues et al. 2025) on interventions to improve nature connection, accounting for and integrating the sociodigital (see Halford and Southerton 2024) and the geopolitical (e.g. Ramutsindela et al. 2020; Gutberlet 2022). This is firmly supported by the evidence outlined in the IPBES Transformational Change Assessment when it states strategies must be synergistic, complementary, customised in bundles and understood as a pathway or process of change.

A seminal systems thinker, Donella Meadows ranked changing paradigms—the assumptions and patterns of thought that ground our worldview—as the most effective place to intervene in a system (Meadows 2008). Currently, however, disproportionate energy is currently being invested on the *least* effective end of systems change—on measurable system features (witness the focus on trying to measure biodiversity) or on compliance reporting—rather than on the design of the system and indeed its intent (Abson et al. 2017; Fischer and Riechers 2019; Dorninger et al. 2020; Richardson et al. 2020a; Riechers et al. 2021a). By identifying paradigm shifts as a deep leverage point, Meadows prefigured what is now an emerging consensus in environmental science policy: beyond the IPBES, the IPCC, United Nations Environment Programme (UNEP), United Nations Development Programme (UNDP), European Environment Agency (EEA) and Convention on Biological Diversity (CBD) all acknowledge the need for deeper cultural change. For example, the EEA’s 2023 report *Existing the Anthropocene? Exploring Fundamental Change in our Relationship with Nature* examines how understanding our deep interconnection with other forms of life could boost motivations to protect nature and accelerate the transformation needed to flourish within planetary limits (Strand et al. 2022), while the UN Human Development Report 2020 states that “Nothing short of a wholesale shift in mindsets, translated into reality by policy, is needed to navigate the brave new world of the Anthropocene, to ensure that all people flourish while easing planetary pressures” (UNDP 2020, p. 24). Elsewhere, the UNDP notes that “Consciousness and mental models are increasingly recognised as the key to unlock systems change” and urges that “It is time to integrate mindsets, values and meaning into work processes, organisational and institutional changes—at the centre of our work. This inner transformation is instrumental to unlock the kind of deep systemic transformation we need, which requires a new vision for building meaning in our lives and our role as a species, as an integral part of the Earth community” (Bovarnick and Legrand 2022). The focus on inner transformation is also reflected by the arrival of Inner Development Goals (IDGs; Ankrah et al. 2023) to complement the Sustainable Development Goals (SDGs) and the Inner Green Deal (Janss et al. 2023) to help deliver the European Green Deal.

Paradigm change in the context not just of biodiversity but the wider polycrisis involves mending our broken relationship with nature, which has at its core the strengthening of a sense of nature connectedness (Richardson 2023). This can be defined, following Schultz (2002), as the extent to which nature is included in one’s sense of self. Expanding, Richardson and Butler (2022, p. 5) explain that “Nature connection is about our relationship with nature—how we think about, feel about, and experience nature. When we feel very close to nature, we recognise ourselves as part of the natural world, and value our relationship with it. We notice nature, seek it out, and feel happy when we are in it”. In this article, we have chosen the terminology of “nature connectedness” for its currency in the literature, but various other closely related if not identical constructs have been used, including connectedness to nature, nature relatedness, love and care for nature, emotional affinity towards nature, dispositional empathy with nature and inclusion of nature in the self (Restall and Conrad 2015). There is now abundant evidence that higher levels of nature connectedness have positive impacts on health, happiness and well-being (Capaldi et al. 2014; Bakir-Demir et al. 2021; Grabowska-Chenczke et al. 2022; Silva et al. 2024).

Given the scale and magnitude of the polycrisis, its heterogeneity and the overwhelming empirical case supporting a view that nature as intrinsically valuable also increases pro-environmental behaviours (Gagnon Thompson and Barton 1994; Mackay and Schmitt 2019; Areias et al. 2023), *compounding* the effects of nature connectedness is vital to deliver systemic shifts. Our focus here on nature connectedness and programme design to achieve compounding effects is not the only answer to the broader question of how societies and cultures can support nature connectedness, but we suggest it might be of greater importance than previously realised. The hope would be of (re)creating positive feedback loops: whereby participating in nature conservation activities increases nature connectedness (Rogerson et al. 2017); as does, in the longer term, restoring nature to enable increased encounters between humans and other species. In aggregate, then, building nature connectedness is a crucial means by which intrinsic value will be ascribed to more-than-human life, thereby pulling one of the most powerful levers for systems change (Riechers et al. 2021b).

## An attentional framework for nature connectedness interventions

**Fig. 1:**
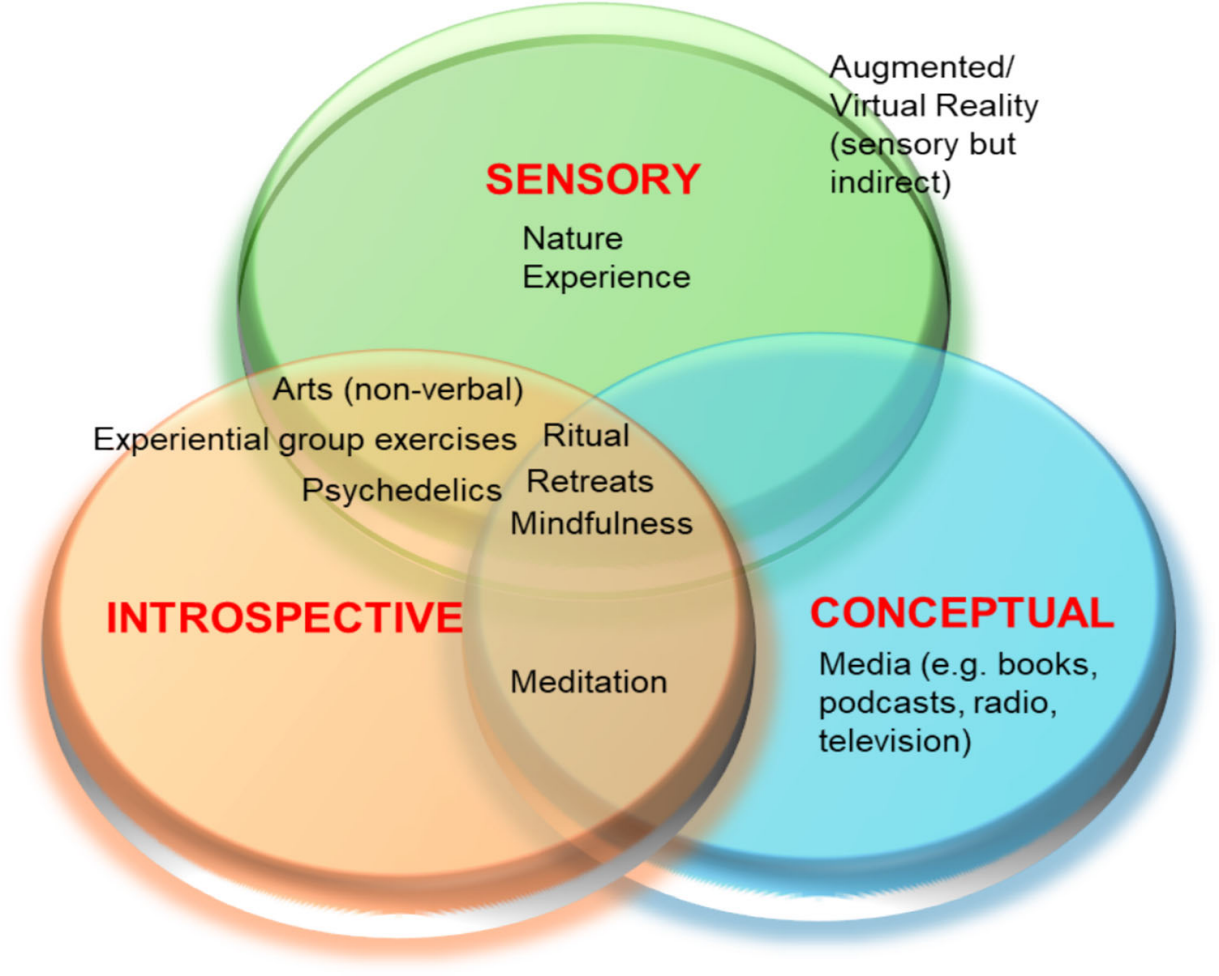
Mapping interventions for nature connectedness according to attentional focus on sensory, introspective or conceptual modes

How do we build nature connectedness? The societal determinants of nature connectedness are complex—studies suggest that childhood experiences, parental modelling, cultural values, socio-economic circumstances, demographic factors and urbanisation may all play a role (Lengieza and Swim 2021; Passmore et al. 2021; Macias-Zambrano et al. 2024; Richardson 2025). It is also important to consider group processes, such as social pressure, support and norms. For example, there is some evidence for the effectiveness of group nature activities (e.g. Lengieza and Aviste 2025), though more studies are needed into these dynamics. However, as we will discuss, various interventions can have a demonstrable impact on individual participants against one of several nature connectedness measurement scales. By “intervention” we mean any activity anticipated to have a positive effect on nature connectedness. In Fig. 1, we map intervention types according to the primary modes by which they engage nature connectedness. These modes refer to attentional direction. In the sensory mode, attention is directed towards contacts with the external world. In the introspective mode, attention is directed towards internal bodily states. In the conceptual mode, attention is directed towards propositional or representational constructs.

We introduce this new framework to help identify an intervention’s attentional directionality and how, through their different modes, they might be organised to concentrate attention and drive transformational change. This is rather different, then, to Shanahan et al. (2019)’s framework, which consists of 27 nature-based health interventions (NBIs), because these are specific facilities or activities themselves such as provisions of gardens in hospitals, outdoor gym equipment or forest bathing, rather than the actual psychological/emotional routes and pathways. Instead, it intersects in different with at least four existing frameworks in different ways, and we consider our framework to be additional, with a usefully distinct lens, rather than being a replacement for others, many of which are very valuable.

First, Lumber et al. (2017) developed their five pathways to nature connection framework from an assessment of Kellert and Wilson’s (1993) nine values of biophilia: utilitarian, naturalistic, ecologistic–scientific, aesthetic, symbolic, humanistic, moralistic, dominionistic and negativistic. As values, these differ from attentional modes, but the five pathways of contact, emotion, beauty, meaning and compassion (see also Richardson et al. 2020a) do relate to the modes in various respects. “Contact”—the one-word label for “contact through the senses”—is equivalent to our sensory mode, although beauty in particular may also be conveyed sensorially. Introspection—especially where directed—can involve all the four non-contact pathways. The conceptual mode can also play an important role as a vehicle for other pathways, especially meaning and compassion.

Second, there are overlaps with Ives et al. (2018), who identify five general types of human–nature connection: the material, experiential, emotional, cognitive and philosophical. The material and experiential are accessed most immediately through sensory attention, the cognitive and philosophical are most commonly accessed through conceptual attention and more directional forms of introspective attention, while the emotional—defined as “feelings of attachment to or empathy towards nature” (Ives et al. 2018, p. 1391)—cuts across all the modes.

Third, we note Giusti et al.’s (2018) comprehensive “Assessment framework for Children's Human Nature Situations” which outlines what to quantify or qualify when assessing environments that might connect children with nature. The framework identified 16 “qualities of significant nature situations” alongside 10 abilities that constitute children's nature connectedness. The attentional modes are evident in many of the 16 qualities: “thought provocation” and “creative expression” tend to be engaged by the conceptual mode, “engagement of senses” and “awe” by the sensory mode and “mindfulness” by the introspective mode. However, the full set of 16 qualities represent a broader category than our focus on forms of attention.

Finally, Sheffield et al. (2022) categorised the interventions in their review according to three dimensions: type of contact (direct vs indirect), quality of engagement (active vs passive) and timing/duration (single session, repeated practice, residential). On types of contact, “direct” would be confined to our sensory mode and specifically experiences in nature. Their distinction in quality of engagement between “active” and “passive” is based on whether instructions were given to participants to be aware of, or engage with, nature. This clearly relates to attention, but not to attentional mode itself. Our framework does not explicitly deal with timing/duration.

Our framework has some broader connections beyond the nature connectedness field. For example, there is a loose mapping to Iain McGilchrist’s (2009) work on how the left and right brain hemispheres engage differently with the world. We note that many neuroscientists suggest McGilchrist over-generalises hemispheric differences into broader “left vs right brain” worldviews and extrapolates to cultural and historical patterns in ways that cannot easily be tested empirically (see, for example, Corballis 2014). However, in a more modest context, the focused attention and abstractions posited by McGilchrist to be favoured by the left hemisphere might be associated with the conceptual-based interventions that we have brigaded together under the heading of “media”, but also with the informational additions provided by AR, and the content of introspective practices like mindfulness or directed meditation. The more global and emotion-linked attention favoured by the right hemisphere might be affiliated with nature experience, psychedelics, undirected introspection and non-verbal arts. It is not a perfect fit, but given the evident heterogeneity of nature connectedness, alignment with McGilchrist’s model helps situate interventions in the context of a broader worldly engagement.

There is also a relationship between our framework and multimodal learning (Bezemer and Kress 2015). Multimodal learning intentionally uses different senses and different parts of the brain, and can be more engaging than, say, learning through text alone, as well as catering for a range of individual preferences. However, it is defined as learning that integrates types of data such as text, audio, images or video, which is a narrower definition of “modes” than the sensory, introspective and conceptual. Also, while multimodal learning can improve information retention, nature connection is not the same as learning. Hence, a multimodal learning lens might help to enrich interventions (especially those with a significant sensory aspect), but it does not necessarily increase nature connectedness.

Our sensory-introspective-conceptual framework is a simplification and there is a big hinterland of scientific and philosophical debate concerning definitions of—and relationships between—these and other modes. They overlap in various complex ways and many interventions may straddle the boundaries. However, it remains a valuable way to map the interventions according to their primary attentional focus. After reviewing the interventions, we will return to questions of potential synergy between the modes when we consider how interventions might be combined under a theory of generating compounding effects within systems.

## Potential for compound nature connectedness effects

To achieve systemic transformational change, we first provide an overview of the field of nature connectedness to position proposals for achieving a *compound effect* where interventions (or events) join forces to cause proportionately larger, cross-domain and persistent shifts (King and Jones 2021). Compound event research is a young field, and challenges in studying compound effects arise because it is “very difficult to generalize insights from individual events, and pooling events to allow for robust statistical analyses needs to be done carefully. Data on impacts are another challenge, as these data often only exist on very limited domains or regions” (Zscheischler 2024, p. 2). Further analytical challenge arises as we are dealing with socioecological systems which are inherently linked systems, “with multiple feedbacks and interdependencies” (Milkoreit et al. 2018, p. 2), and studying them separately is problematic (and potentially counterproductive) (ibid). Interventional studies and experimentation will always need to reduce complexity to isolate variables for testing. By examining evidence that could potentially build towards compounding effects, we are seeking to identify how multiple, cross-system interventions across our attentional modes framework can be leveraged.

Our literature review covered peer-reviewed publications written in English and published before 31 March 2025, with nature connectedness as an outcome variable, whether or not an established nature connectedness scale was used. Searches using a UK university database encompassed Web of Science, Scopus, JSTOR, DOAJ, MPDI and others, using the keywords “nature connect*”, “nature relatedness”, “nature awareness”, “connection to nature” and “connection with nature”. In order to ensure coverage of interventions, the above terms were combined where necessary with "meditation", "mindfulness", "arts", "psychedelic*", "virtual reality" and "augmented reality". We next summarise the evidence underpinning these broad intervention types, before discussing the potential for combinatory approaches.

There is an increasing body of research on the different interventions for nature connectedness, including meta-analyses and review papers. The field is attempting to establish and test theories of nature connectedness empirically, so understandably knowledge still remains siloed and fragmented across different disciplines, with research weighted towards certain intervention types (especially nature experience). This paper attempts to fill the gap by combining a wide range of interventions with an exploration of compound effects.

Others have laid the groundwork. For example, Lumber et al. (2017) assess the pathways to nature connectedness using the nine values of biophilia as a basis (Kellert and Wilson 1993). They conclude that “Researchers and practitioners interested in facilitating nature connectedness and its associated benefits should focus specifically on activities that involve contact, meaning, emotional attachment, or a compassionate relationship with nature that includes engaging with nature’s beauty” (Lumber et al. 2017, p. 21). They were introducing the pathways for the first time, so understandably tested them against a single intervention—being in nature. Richardson et al. (2020a) highlighted the utility of the pathways and how they could be used in more systematic ways. As one might expect, the early adopters of the pathways approach used them to design nature experiences, and the paper focuses on The Wildlife Trust’s “30 Days Wild” campaign. However, the pathways have since been applied to buildings, schools and meditations.

Recognising that a network of factors influence sustainability outcomes, Lengieza et al. (2024) combine the pathways framework with the lens of emotions. They find that awe, inspiration and love are all important and central to nature connectedness experiences, and reinforce other research on the significance of meaningful and deliberate engagement. Interestingly, when participants were asked for their notably positive nature connectedness experiences they reported experiences in nature rather than other possibilities such as mindfulness, psychedelics and the arts. This is an interesting finding, but should be considered in the context of the evidence we discuss below on the potential impact on nature connectedness of these other intervention types.

There are some wider-ranging reviews of nature connectedness interventions. Ives et al. (2018) discuss art, religion, meditation and community gardening, while Barragan-Jason et al. (2022) cover nature experience, mindfulness and education. Of the 36 studies meeting the criteria for Sheffield et al.’s (2022) meta-analysis, 27 involved “direct” contact through exercise or other activities in a variety of outdoor settings or otherwise engaging with nature in particular ways, while the remaining nine involved images, videos, audio or guided imagery/meditation practices. Each study is less comprehensive in the range of interventions covered than the set we consider here, and none considers combinations in depth, but this is to be expected, as comprehensiveness is based on the interventions others have designed, tested and published and suitable for inclusion in a meta-analysis. Riechers et al. (2021a) discuss combinations of leverage points, though in abstract terms.

We note also the relevance of “The Work That Reconnects” (WTR; Macy and Brown 2014; Jones and Johnstone 2024), even though it was developed too early to be informed by the blossoming of the academic study of nature connectedness. WTR is a groupwork model combining psychological and philosophical teachings with experiential practices, aimed at helping people to explore the predicament of the planet and their role in its transformation. The experiential practices are usually mapped to four stages called “gratitude”, “honouring our pain for the world”, “seeing with new and ancient eyes” and “going forth”. Some of the practices build psychological capacity while others are designed for frequent reuse. While WTR is not explicitly structured to achieve compounding effects, the intention is implicit in how the elements are intended to form a transformational whole.

In sum, there is a growing body of sophisticated work on nature connectedness, but some interesting areas remain unexplored, as others have noted. For example, Sheffield et al.’s (2022) analysis “confirms that carefully designed interventions can deliver sustained increases in nature connectedness” but that “although options for fostering connectedness are available now, the range is limited”. They emphasise “the need for examining a wider range of nature engagement activities, greater understanding of factors leading to increases in nature connectedness, design and testing of practices for sustained nature connection, and initiatives that engage people with nature, create conditions for nature connection, and encourage repeated nature engagement activities” (Sheffield et al. 2022, pp. 1, 19). A few studies have discussed more than one intervention type, but it is less common to compare a very wide set or to explore compound interventions in detail. This is perhaps to be expected. As Spaling (1994) notes, “cumulative effects assessment requires a temporal scan of long duration and geographic representation at various scales” and rightly, most studies cited here are tightly controlled and focused in scope. They are, however, “building up the empirical base” required for “rigorous analysis of cumulative effects” (Spaling 1994, p. 249). This invites further investigation on synergies between them and potential combinations that could produce significant shifts in nature connectedness.

## Evidence on effectiveness of interventions

In this section, we provide a narrative review on the effectiveness of several individual intervention types in order to set the stage for an exploration of compound interventions.

### Nature experiences

It is well established that nature connectedness promotes a range of physical and mental health outcomes (e.g. Capaldi et al. 2014; Twohig-Bennett and Jones 2018; Pritchard et al. 2020), but there is now abundant evidence that it can also foster pro-environmental attitudes and behaviours (Mackay and Schmitt 2019; Richardson et al. 2020b). Nature experiences specifically are associated with such attitudes and behaviours. A recent review paper found direct experiences of nature to be positively associated with a range of pro-environmental actions including recycling, energy conservation, green purchasing and conservation volunteering (Soga and Gaston 2024). Another study found that those who described nature in experiential or complex terms were more likely to participate in environmental volunteering, citizen science, litter picking and community gardening than those who used descriptive terms (Hatty et al. 2022).

Some studies investigate which specific aspects of those nature experiences have the greatest impact on attitudes and behaviours. In theory, influential variables could include the kinds of habitats and ecologies experienced, the richness of biodiversity and the cognitive states and philosophies held, as well as temporal variables such as duration, frequency, seasonality and time of day/night. Moreover, this range of potentially influential variables—and their near-infinite number of combinations—may have different impacts according to various characteristics of the individual or group concerned. A “one-size-fits-all” approach to nature experience interventions may therefore be less effective.

However, one factor which does emerge, and is perhaps more important than the characteristics of the environment, is what people are doing while in nature (Richardson 2023). Studies in aggregate suggest that combining nature experience with other activities can boost nature connectedness so long as those activities are related to nature exploration, engagement or appreciation (Martin 2004; Lumber et al. 2017; Pocock et al. 2023).

Finally, it is worth noting the concerning dynamic that as nature is increasingly destroyed, this drives a negative feedback loop whereby the environments encountered are impoverished, if they are accessible at all. A shifting baseline situation thereby arises where there is less nature connectedness and hence fewer pro-environmental behaviours, which then facilitates further destruction (Balázsi et al. 2019; Riechers et al. 2020; Hamlin and Richardson 2022; Oliver et al. 2022).

### Augmented and virtual reality

Digital, artistic and other forms of representation—whether visual, auditory, olfactory or other—involve a layer of distancing from “direct” nature experience. Several studies comparing virtual reality representations with an equivalent physical experience suggest that impacts on positive affect and mood, for example, are greater for the latter (Browning et al. 2020; Markwell and Gladwin 2020; Reese et al. 2022). However, there is still some evidence that such representations of nature—and specifically AR and VR—have the potential to promote not just mental well-being and pro-environmental behaviours but nature connectedness too. This may depend on factors such as the design, the technological substrate and the duration of exposure (Klein and Hilbig 2018; Leung et al. 2022; Brambilla et al. 2024; Loy et al. 2024; McKeever et al. 2024).

There are many potential variations in AR/VR nature experiences that could influence their effectiveness in building nature connectedness. In their review paper, Spors et al. (2023) acknowledge the variety of virtual human–nature interactions being produced, while critiquing the ways in which designers unavoidably incorporate their own values, judgements, biases and understandings in terms of both what nature is and what it should be like. Hence, opportunities lie not only in technological improvements in the realism of the immersive experience, but in moving beyond the current dominance of simple and positive experiences to more complex, challenging ones. This would afford a richer and more realistic set of virtual and augmented experiences, better able to convey nature’s grandeur and mystery, rather than purely provide a source of peace or escapism. There are also opportunities to give a sense of other times (whether past or present), to aid the imagination by vivifying environmental risks and opportunities (including helping with shifting baselines) and to assist in adopting non-human perspectives.

In aggregate, research suggests that physical experience has greater and more long-lasting impacts than virtual reality, but that the latter is still an improvement over no nature-related experience at all, or over virtual reality experiences that omit nature. This suggests a valuable role for AR/VR and other representations where immediate nature access is difficult, but should not come to be seen as a viable replacement, either in terms of the experience itself or—more alarmingly—that the existence of a digital version makes the real thing more dispensable (Beauman 2022). There are also risks around attention capture, unhealthy advertising and the various environmental costs of electronics.

### Mindfulness

Both mindfulness and meditation feature in the recent emphasis on the importance of inner transformation for sustainability (Bristow et al. 2022, 2024). They have much in common but are better understood as separate interventions. Mindfulness is an intentional, non-judgmental attentiveness to the present moment (Kabat-Zinn 1996), involving conscious awareness of feelings, thoughts and sensations. Hence, in Fig. 1 we suggest it lies across introspective, sensory and conceptual modes as a form of introspection that often entails focused attention on what is received by the senses as well as on concepts entering the mind. Meditation, on the other hand, while also an introspective practice and sometimes involving focusing on a concept, tends to downplay sensory engagement.

Mindful nature walks, attentional practices focused on ecological features and similar mindfulness activities such as forest bathing have become more popular in recent years and have been shown to help extend compassion towards nature (Kurth et al. 2020; Ramstetter et al. 2023). Mindfulness practices align strongly with the pathways that Lumber et al. (2017) propose, including noticing nature, appreciating its beauty and nurturing compassion. They note that it is not merely time in nature but the quality of engagement that matters, and this is something that mindfulness can support. The relationships may be two-way: nature experiences may themselves help to develop a sense of presence and mindful non-evaluative attention, while mindfulness can allow people to engage more fully with nature experiences (Schutte and Malouff 2018). Other studies indicate that connection to nature and mindfulness can operate synergistically to enhance mental well-being and pro-social values (Howell et al. 2011; Wolsko and Lindberg 2013). This effect has been found in both wilderness contexts and urban settings (Nisbet et al. 2019; Farkić et al. 2020). On balance, mindfulness would appear to have a positive role in building nature connectedness, even though Richardson and Sheffield (2015) find “reflective self-attention” to be a more reliable predictor of nature connectedness than “mindful attention”.

Beyond nature connectedness, many studies demonstrate a relationship between mindfulness and pro-environmental behaviours (e.g. Wamsler and Brink 2018). Moreover, research on the so-called Mindfulness-Based Stress Reduction reveals that its impacts on mental health and well-being are greater when practised in nature than in indoor or built environments (Choe et al. 2020). Given the role of nature experience in enhancing nature connectedness, it is likely that mindfulness in a nature setting will contribute more to nature connectedness than practising it elsewhere.

### Meditation

While mindfulness in a nature setting may be optimal, there are probably still nature connectedness benefits regardless of setting, and this is perhaps even more apparent in the case of meditation. Interoception is the sense allowing us to be aware of the physical and emotional processes in our bodies, and meditation is known to enhance it. Branham (2024) finds that interoceptive awareness predicts nature connectedness, that secure attachment to nature explains this relationship, and that these predict both well-being and pro-environmental behaviours. This suggests that interpersonal relational processes essential for human bonding also occur in nature bonding. The study also reports that, among the interoceptive processes, emotional awareness most significantly predicts nature connectedness, suggesting that the more aware a person is of the connection between inner bodily sensations and emotions, the more likely they can bond with nature. This may explain why practices such as meditation, known to enhance interoceptive awareness, can enhance nature connectedness regardless of setting.

The “content” of the meditation may be significant. For example, explicit meditative engagement with, say, water or air can help to disrupt modern distinctions between humans, things and the environment (Carvalho and Riquito 2022). Experiments also suggest that both mindfulness meditation and loving-kindness meditation can increase not just nature connectedness but sustainable decision-making (Adventure-Heart and Proeve 2017; Engel et al. 2020). There is little research on sequences of meditations and which might be most effective, but we note that a Council of All Beings ritual moves from mourning to a cosmic story of interconnectedness and then each participant preparing to represent a particular species (Seed et al. 1988). Future research might explore the impact on nature connectedness of various potential meditative sequences.

### Psychedelics

There is some evidence that careful use of psychedelics—in particular psilocybin, LSD and ayahuasca—can increase nature connectedness through dissolution of a sense of ego (Nour et al. 2016; Forstmann and Sagioglou 2017; Lyons and Carhart-Harris 2018; Kettner et al. 2019; Aday et al. 2024). Participants in experiments report sentiments such as:

- “Before I enjoyed nature, now I feel part of it. Before I was looking at it as a thing, like TV or a painting. [But] you’re part of it, there’s no separation or distinction, you *are* it”.
- “I felt like sunshine twinkling through leaves, I *was* nature” (Watts et al. 2017, p. 534; emphases in the original).

There is also evidence suggesting short-term interventions can be beneficial for treating depression and obsessive–compulsive disorders (Carhart-Harris et al. 2018).

There may be a link from psychedelic-raised nature connectedness to pro-environmental behaviours, which would be expected given the aggregate weight of evidence associating nature connectedness (from whatever source) with pro-environmental behaviours. However, there is evidence that self-reported values and beliefs of the users of psychedelics reflect more concern for the environment than in non-users or users of other substances (Lerner and Lyvers 2006; Studerus et al. 2011; Forstmann and Sagioglou 2017). There has also been a retrospective survey of 150 psychedelic users, all of whom reported increases in nature connection following use, but with 66% also stating an increase in their environmental concern. Indeed, 16% of participants changed their careers following psychedelic use to work they deemed more environmentally oriented (Luke 2019).

In the literature on psychedelics, the variables beyond dosages and frequencies—“set” (psychological context) and “setting” (sociocultural context)—are known to be a key determinant of experiences. A number of recent studies suggest that the previously reported increases in nature connectedness and associations with pro-environmental mindsets are not reproducible (Forstmann et al. 2023; van Elk and Fried 2023; Orłowski et al. 2024). Some initial work suggests that psychedelic use within a nature setting, rather than in a clinical environment, might be advantageous for nature connectedness (Hartogsohn 2017; Gandy et al. 2020), but more research is needed. In aggregate, there continue to be challenges disentangling whether associations between psychedelic use and nature connectedness are merely correlative or causative. We also caution that there is also increasing evidence of adverse effects from psychedelic use, especially due to inadequate set and setting factors (Simonsson et al. 2023, 2025).

In terms of content, psychedelic activity may not only have different effects depending on pre-existing worldviews, but also influence the worldviews themselves beyond nature connectedness (Lyons and Carhart-Harris 2018). Finally, multiple experiments have shown that nature connectedness effects can remain months if not years after the acute experience (Pahnke 1963; Doblin 1991; Studerus et al. 2011; Lyons and Carhart-Harris 2018; Kettner et al. 2019).

### The arts

A large literature exists on nature and the arts, but less on the relationship between the arts and nature connectedness specifically. As discussed, it is not merely being in nature but what we do there that can be significant for growing nature connectedness, and the arts have the ability to increase and diversify forms of engagement, change patterns of thought and leave an enduring impression (Raatikainen et al. 2020).

Whether for verbal or non-verbal arts, there can be challenges in collecting, interpreting and representing arts-related data. Where studies do exist, some are not particularly systematic, and tend not to use the same measurement methods for connectedness as some other interventions. This is not necessarily a shortcoming of those approaches; indeed, the so-called arts-based research (ABR) methods have been increasing in popularity because they offer opportunities for richer and more nuanced accounts of nature connectedness, encompassing aspects such as embodied and sensory experience that may have previously been neglected (Muhr 2020; Niemelä et al. 2023). Regardless of whether an ABR method was used, there is mounting evidence that the arts have a role to play in more active nature awareness and engagement, and in some cases nature connectedness specifically. This evidence exists for both adults (Riechers et al. 2019; Renowden et al. 2022) and children (Gilbertson 2012; Gray and Birrell 2015; Gray and Thomson 2016; Moula et al. 2022, 2023; Majid et al. 2023).

Some studies have used non-verbal arts to develop some form of empathy with other species, varying in the depth of engagement they involve. At one end of the scale sit interventions such as field recordings and related “art–science alliances” (Taylor 2024), feeling vibrations akin to those endured by benthic species due to anthropogenic activities (Gonçalves and Penha 2021), and experiencing a “Coral Empathy Device” (Carvalho and Riquito 2022). The more engaged end of the scale includes using drama to build nature connectedness through exercises in “becoming” all sorts of other creatures (Pearce 2024) and musical “jamming” with cetaceans, birds and other species (Mathews 2008). More generally, there is evidence that playing or composing music outdoors can lead not just to nature connectedness but to extraordinary, “spiritual” experiences (Adams and Beauchamp 2019; Arbuthnott and Sutter 2019).

In other cases, arts interventions seek to increase connectedness less with other species and more through deeper engagement with habitats and landscapes. This can be with physical habitats, such as designing trails to entice curiosity (Spiller 2024), but also their artistic representations (Tribot et al. 2022). Hence, while AR/VR offer one route by which we can access representations of inaccessible environments, (non-digital) arts open other avenues.

### Media

The final type of intervention we assess involves conveying conceptual content about nature more directly. To some extent, this overlaps with the arts, but here, under the umbrella term of “media”, we include television, radio, podcasts, complex verbal arts like theatre and opera, and books (whether fiction or non-fiction, and everything from poetry to textbooks). There is little research on the value for nature connectedness of these interventions, but scholars have attempted to study the value of conceptual content. For example, Lumber et al. (2017), in their evaluation of the pathways to nature connectedness, found the knowledge pathway to be ineffective: those who engaged with nature through the naming of species did not see an increase in their nature connectedness. Similarly, Barragan-Jason et al. (2022) found that environmental education had no significant effect.

However, other frameworks include conceptual content in their understanding of what constitutes nature connectedness. These include Nisbet et al. (2009), who in describing the Nature Relatedness Scale argue that nature connection involves not just emotional affiliation to nature and seeking regular contact with it, but comprehending the importance and interconnectedness of all facets of nature. Aligned with this expectation, a before-and-after nature connectedness survey associated with a non-fiction book was found to significantly increase scores using the Nature Relatedness index, NR-6 (Nisbet and Zelenski 2013; Oliver et al. 2020, Oliver unpublished data). There are then the five general types of human–nature connection identified by Ives et al. (2018), which extend beyond the material, experiential and emotional to the cognitive and philosophical, with the emotional and philosophical reportedly holding the greatest transformative potential. These are consistent with substantial anecdotal evidence that many people believe their nature appreciation (and so potentially their nature connectedness) has been boosted by facts about nature. They find such information a source of wonder and fascination and, as Wendell Berry stressed, wonder and fascination about nature may be requisite for its preservation (see McKibben 2022, p. 179), as well as a motivation to engage in direct nature experiences.

The type and form of knowledge tested by Lumber et al. (2017) were intentionally narrow in an attempt to isolate this variable. Further research is therefore needed on the role of different facts in enhancing nature connectedness, on how those facts are combined, on narrative form and on the various genres and media by which they can be conveyed. It may be that conceptual information—including scientific information—engages some of the pathways that Lumber et al. *did* find to be effective for nature connectedness, such as beauty, meaning and symbolism. Future research should also assess individual differences in the affectivity of the information, how effectiveness varies according to the quantity and frequency of such information, and the advantages and disadvantages of combining the information with other types of intervention.

In summary, looking across the evidence reviewed on nature connectedness interventions, the areas to explore for achieving compounding effects can be distilled as the diversity and quality of perceptual content, repeated and sustained immersions, purpose and intent while connected, and the potential to facilitate feelings of transcendence. These areas complement Lumber et al.’s (2017) nature connectedness pathways of *contact, emotion, beauty, meanin*g and *compassion* as they concern the structure of the experience, rather than the qualitative character of the experience, and therefore provide options for strategic arrangements of interventions, orchestrated specifically to achieve compound effect.

## Understanding compound effects

As our review highlights, an array of interventions are proved to increase nature connectedness and the pathways are well developed, even if not mainstreamed in policy. In this section, we propose a theoretical outline of how to achieve compound effects to catalyse system transformation (Gunderson and Holling 2002; Yaman and Polat 2009), premised on the hypothesis that amplification of nature connectedness could support more radical systems change.

Compounding as a phenomenon or concept has been researched across many disciplines (summarised in Table S1). For example:

- Ecology: Kleinman et al. (2019, p. 2), in reviewing compound disturbances in forest ecosystems, describe compound effects as “…the combined effects of multiple disturbances… altering the rate or trajectory of forest recovery…; the interaction is *amplifying”* (our emphasis). And as Buma (2015) outlined, compound interactions of disturbances require *two different mechanisms* (e.g. low regenerations of seed dispersal and serotinous species due to altered burn characteristics) to alter ecosystem resilience.
- Information science: Li et al. (2022) find compound adverbial attacks, combining two attack methods that target the same system node, are more effective, as the [attacked] system’s response is compromised. Using “a systematic analytical technique for modelling and analysing compound information warfare strategies”, Kopp (2005, p. 7) illustrates the *combinatory leverage of multiple activities* (e.g. a phoney radio signal, a cyber attack, destruction of devices, targeted propaganda, etc.) on a specific node (or “victim”), and the difficulty in effective counters due to its multifaceted nature.
- Risk: Within the risk literature, the concept of compound effects is routinely operationalised (Schlosser et al. 2023) and understood as “two or more relatively rare and high-consequence events that co-occur in time and space, amplifying their effects” (Klasa et al. 2025, p. 1), leading to multiplicative effects and overwhelming system actors. Similarly, Linkov et al.’s (2024) modelling of the Late Bronze Age Collapse highlighted that only the simultaneous loss of cross-system regional nodes (e.g. two or more nodes within the political and trade systems) led to cascading failures.
- Organisational theory: In an instructive empirical study of organisational restructures (cf. reconfigurations) based on complex adaptive system theory, Girod and Whittington (2015, p. 1532) conclude that while “…discrete perturbations [can] eventually [lead] to discontinuous change…[a]ccumulated incremental change is only likely to trigger discontinuous change in conditions of disequilibrium. Ordinarily, then, it is bursts of change that are more likely to lead directly to escalation”. Similarly, Willcock et al. (2023), reporting on computer simulations of ecological collapse, find that “real-world tipping elements are more likely to be driven by multiple, fast drivers and extreme events”.
- Economic theory: Here, the idea of compounding effects—reinvestment of interest gained on top of existing investment—has a long history. Tracing 4,00o years of economic thought, Hudson (2000, p. 345) documents how the “mathematics of debts mounting up compound interest tend[ed] to overwhelm the economy’s ability to pay”, further demonstrating that accumulation, if targeted, can generate significant amplification and disrupt (or cause to falter) incumbent structures.
- Military strategy: The concept of achieving compound effects appears extensively in military strategy in various doctrinal forms. Compound Warfare (Huber 2002), specifically, can be defined as “conflicts with regular and irregular components fighting simultaneously under unified direction” where “complementary effects are generated by its ability to exploit the advantages of each kind of force and by the nature of the threat posed by each kind of force” (Hoffman 2009, p. 3).

Drawing from these findings, we summarise that compound effects in a system are produced when (a) multiple events, (b) of more than one type, (c) impact nodes susceptible to disruption, at (d) a speed or scale that (e) compromise or overwhelm the system’s ability to recover or mount a defensive response. We conclude that, given the cross-disciplinary consistency in detailing the characteristics of compounding phenomena, compound effects are most likely to occur when every element is activated.

## Achieving compound effects in nature connectedness

Informed by this diverse literature on compounding effects and, specifically with regards to nature connectedness, building on Richardson et al.’s (2020a) work outlining system leverage points to achieve nature connection at societal scale, we present a stepwise approach (see, for example, van Ginkel et al. 2022) for the strategic arrangement of nature connectedness interventions that should, theoretically, achieve a compound effect. Though the weight and complexity of the evidence on interventions varies, a reasonable null hypothesis is that the effects are additive, and unlikely to be antagonistic (as supported by Pocock et al.’s (2023, p. 591) finding that combining citizen science with noticing three good things in nature (3GTiN) “engaged the pathways to nature connectedness at least as strongly as the highest scoring of citizen science or 3GTiN individually”), and that it is the systemic application around the compound elements we highlight above that generates effect. It is worth clarifying that our theorisation is built on the evidence that compound effects can bring about systems change, but in a real-world setting (e.g. an advanced industrialised economy) this would entail (and require) dissolution of the existing system (i.e. a regime change). In the literature we reviewed above, this is predominantly studied from a perspective of reduced existing system resilience, an inability to recover and thus collapse, rather than “transformation”. While we make no moral or normative assessments here, the system characteristics of collapse and transformation can be considered agnostic for our purposes.

### Step 1: Target susceptible nodes

A node in this context for enhancing nature connectedness could be an individual person, a village, town or city, a region or a particular institution (Moodie and Wheelahan, 2024), organisation or network itself, where they have a high centrality measure (e.g. Ghalmane et al. 2019) and at least some measure of susceptibility (Laursen and Feur, 2022; Hu et al. 2022), e.g. personal crisis, economic hardships (e.g. Lee et al. 2015), strong countercultural activist base, etc. Nodes of susceptibility are therefore types of systems leverage points, following Meadows’s (1999) definition (widely cited in nature connection literature), but are further distinguished by their (in)capacity to withstand, resist and recover from effects. Leverage is achieved by exploiting weakness and countering/suppressing recovery, where recovery is a return to a near-equilibrium state (O’Neill 1998), i.e. the status quo of diminished nature connection. For example, considering ideological orientation to the dominant social paradigm (DSP) (Bogert et al. 2022) and using Fagerholm’s (2016, p. 151) conceptual typology of ideology, only particular subversive ideologies that “reject and consequently, combat” the industrialised DSP would present as a characteristic of susceptibility for our purposes. The ability to achieve compound effects of nature connectedness here is leveraged through the susceptibility of ideologically predisposed individuals and groups to replace “…one interpretation of an environment and a prescription as to how that environment should be structured” (Denzau and North 1994, p. 4) with another, and then reject and combat (re)emergence of the previous ideology (e.g. industrialised capitalism) through collective action (van Zomeren et al. 2008).

### Step 2: Assess and prevent counter-response

A counter-response can be defined, for present purposes, as the means, method and material used to recover and re-establish normal system functioning, passively or actively. In the context of nature connectedness, at different scales (from individual to societal), counter-responses are the anthropogenic pressures that re-assert and intensify following a nature connectedness intervention, and thus, over time, are likely to increase the dissipation rate of increased NCI scores. For instance, as the strongest interventions for nature connectedness involve sustained direct experience with diverse natural settings (e.g. being in ancient woodland, with meadows and ocean views, or a five-day wilderness immersion without phones), when coming out of these settings, most people are likely to return to an urban home environment (increasing disconnection, for example, Kowarik et al. (2025); Speller and Twigger-ross (2009)), be exposed to digital and broadcast media (reinforcing materialist, consumerist behaviour, e.g. Hartmann et al. (2023)), and realign with dominant social practices (e.g. Eastwood et al. 2023) and declining environmental baselines (Soga and Gaston 2024). But this is not isolated to wildspace–urban transitions; even if we sustain experience in naturally diverse settings, the mere presence of other humans is enough to reduce nature connectedness, as Lengieza et al. (2025) have recently shown.

Beyond passive and semi-passive anthropogenic pressures, more indirect responses aimed at stabilising the status quo (again, at difference scales: individual to societal) may need to be prevented, such as increasingly draconian criminal legislation targeting climate and environmental protests (e.g. Falcone et al. 2020), emergency legislation and environmental target backsliding (see McLaren and Corry 2023), and mainstream narrative frames that undermine pro-environmental action (e.g. Falcone et al. 2020). Further still, direct counter-responses to pro-environmental behaviour linked to deep nature connectedness (Mattijssen et al 2020; Hanaček et al 2024; Soga and Gaston 2024) may even appear in the form of physical violence, up to and including massacres, serial killings and assassinations (Tran and Hanaček 2023). Anthropogenic environment pressures, indirect and direct responses, unless prevented or at least suppressed, will almost certainly negate nature connectedness interventions from compounding. We propose that assessing and preventing systemic conditions that may exacerbate the dissipation rate of nature connectedness following interventions is a key research area.

### Step 3. Deliver successive and diverse interventions at speed and scale

Speed and scale of nature connectedness interventions can be simply conceived as (1) *speed*: the time between interventions and before anticipated effects dissipate, and (2) *scale:* interventions that impact through different mechanisms (or attentional modalities) and/or spatial scales, to achieve a form of envelopment (Frers 2007, p. 34) of nature connection. Speed and scale focus on the temporal–spatial dynamics, at different scales (from individual to societal), of compounding effects of nature connectedness. In our theorisation, speed then implies shortening the time between interventions and scale implies a range of interventions at different spatial spaces, across a range of modalities (e.g. within a wetland, in an office, at home, through nature poetry, VR nature experience and ritual) to build synergies (e.g. Djernis et al. 2019).

We can speculate that nature connectedness interventions should be deployed (1) at a speed sufficient to maintain nature connectedness (before effect dissipation) and (2), fast enough to outpace counter-responses (step 2), targeting people, places and systems that have high centrality measure (step 1). For example, following a high-impact invention—such as a five-day wilderness immersion without phones—and assuming a conservative effect dissipation rate of one month, a second intervention, using a different modality in a different space (e.g. social VR Celtic rainforest experience, in the home), could be deployed within the first two weeks. This may meet step 1 (target susceptible nodes), but it would be unlikely to address step 2 (assess and prevent counter-response), since within minutes or hours of the immersion, on the drive home, anthropogenic pressures resume: adverts, radio programmes, social media and ultra-processed food, friends and family, almost immediately start to reduce nature connectedness. As many papers have noted, undergoing a particular intervention increased the motivation of participants to explore or re-engage with other interventions. For compounding effects, it seems necessary to act before dissipation. Thus, capitalising on this motivation, successive interventions should follow quickly after the initial exposure.

There are many ways to sequence and coordinate interventions, but the principles of speed and scale are theoretically of central importance to achieving compounding effects. It is therefore necessary to consider which intervention types afford connection in different places (e.g. the home) and in ways suited to those places (e.g. TV programmes); for example, van Rompay et al. (2023, p. 8) found VR experiences of spacious landscapes increases nature connectedness, providing an effective means “to stage immersive nature experiences” where direct access is restricted. Our sensory-introspective-conceptual framework (Fig. 1) can help inform intervention selection.

Speed and scale alone are unlikely to be sufficient to achieve decisive compound effect and thus transformational change. Take, for example, the combined “shock and awe” and Blitzkrieg of Coalition Forces in the 2003 Invasion of Iraq, which dismantled the incumbent government in under 28 days (a form of transformation), often noted for its speed. As Biddle (2007) writes, speed, while often considered as central to its success, was not sufficient, and it was the Iraqis’ ineffectiveness and weakness that compounded Western advantage. This, again, highlights the importance of leveraging speed and scale of interventions against vulnerable nodes (step 1) and working with anthropogenic counter-response dynamics (step 2). For enhancing nature connectedness, therefore, selection of interventions should be cognisant of, and integrate with, measures that prevent or inhibit these pressures. Speed and scale, while appearing across disciplines as a key characteristic in compounding effects, are perhaps the least explicitly studied, and therefore open up a number of important research questions into the effects of the temporal–spatial dynamics of nature connectedness (e.g. effect dissipations rates, e.g. Spano et al. 2023).

**Fig. 2:**
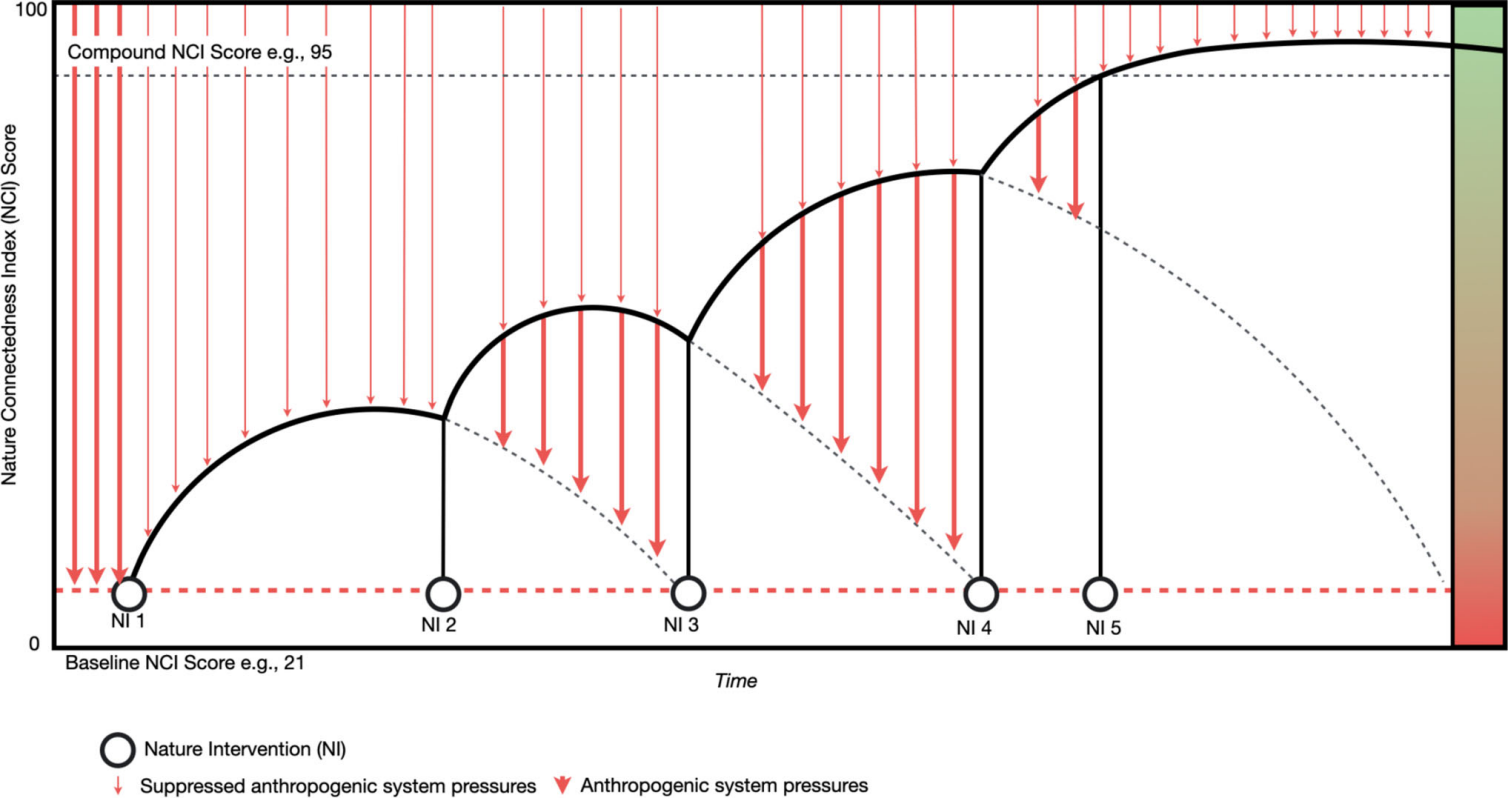
Stylised illustration of compounding nature connectedness interventions over time that suppress anthropogenic pressures and counter-effect dissipation rates. From left to right, this figure shows how a nature intervention (NI 1) improves the baseline NCI score but then, without further intervention, anthropogenic pressures (thin red downwards arrows) degrade the NC effect, and intensify if not countered (thick red downwards arrows). Theoretically, compounding interventions (2–5), reach significantly higher NCI scores and inhibit the influence of anthropogenic pressures

Following these steps presents a simple, evidence-based procedure for strategies and programmes to (a) improve nature connectedness and (b) achieve compound effects in support of more radical systems change, from individual to societal levels. Figure 2 provides an illustration of our theorisation.

## Conclusion

Our review of nature connectedness interventions shows that while some are shown to be partly effective, further research is required into how to amplify their effects to achieve transformational change. Reviewing systems theory from across different disciplines and fields, we distilled the system characteristics that generate compound effects which can contribute to transformational change. Compound effect is achieved when (a) multiple events, (b) of more than one type, (c) impact nodes susceptible to disruption, at (d) a speed or scale that (e) compromise or overwhelm the system’s ability to recover or mount a defensive response. This constitutes a theoretical outline for achieving compounded nature connectedness.

Programme design should consider the most effective sequencing for different interventions. For example, while interventions to convey nature/ecological risk are beyond the scope of this paper, we recommend that the integration of conceptual material—and potentially direct experience—of the realisation of current ecological risks should be explored. Such material and experience is often used at the outset of communication and engagement activity to create a “burning platform”, but this must be done with care, as individuals vary in their tolerance for “negative” information and challenging experiences. However, perhaps later in a sequence, this may bolster nature connectedness and galvanise action in terms of what is being lost, and might be lost in the future, especially if it can be potently embodied in new ways. A second question around sequencing refers to the more conceptual content. Here we recommend further work into how it can be included less as detached material and more as an integrated part of other interventions, recognising that in some cases it can enrich those experiences while in other cases those less conceptual interventions might be more effective in a simpler or more flexible form. Further research is needed, then, to understand the effectiveness of various sequencing options on nature connectedness.

Such research on effective sequencing could form part of a broader evidence-based monitoring and evaluation programme to explore different combinations of interventions. Combinations should be evaluated over different timescales, such as over several days, weeks or months, and, given that many participants have very limited training time and budgets, even over a one-day blend of conceptual and introspective activities carried out in a natural setting. We suggest using an established nature connectedness scale to assess pre- and post-programme nature connectedness, in addition to assessing ultimate pro-environmental attitudes and behaviours, but that this should be complemented by qualitative approaches, especially in areas such as the arts where such methods have generated additional insights. In theory, programme assessments could be carried out for current programme or training providers using different blends of interventions. However, few organisations currently have such a mixed programme, the numbers of participants may not be statistically significant, and there are likely to be sensitivities in publishing results. Hence, systematic testing may require a new programme or training package—whether or not at an existing organisation—which can trial combinations and sequences in a systematic way, and perhaps with the funding necessary to enable significant levels of participation. Alongside other research into combinations of interventions, this would be valuable for developing the kind of training and development approaches that may be critical for genuine sustainability transformations.

## Supplementary Information

Below is the link to the electronic supplementary material.

Supplementary file1 (PDF 148 KB)
