# Supplementary material for: Catalysing transformational change through compound nature connectedness interventions
Source: Ambio. 2025 Dec 31;55(8):1677–96. doi: 10.1007/s13280-025-02328-0 (PMC13319648; doi:10.1007/s13280-025-02328-0)
Supplement: Supplementary file 1 — Supplementary file1 (PDF 148 KB) [file 13280_2025_2328_MOESM1_ESM.pdf]

Electronic Supplementary Material: Title Page

Title: Catalysing Transformational Change through Compound Nature Connectedness Interventions

Journal: Ambio

Authors: Matt Pritchard, Philip Tovey, Phoebe Tickell, Tom H. Oliver

Affiliation of Corresponding Author: University of Reading, UK

Email Address of Corresponding Author: m.c.pritchard@reading.ac.uk

Table S1. Compound phenomena described by discipline/field and their primary methods

**Discipline**	**Description**	**Primary methods**	**References**
Socio-Ecological System (SES)	The combined effects of multiple disturbances altering the rate or trajectory recovery	• Ecological modelling<br>• Observation	*Cioffi-Revilla (2016); Kleinman et al. (2019); Zscheischler et al. (2018) Willcock et al. (2023)*
Information Science and Security	Overloading adversary's capacity (physical or mental) to adapt to the new situation, collapsing their ability to carry on	• Network modelling<br>• Adversarial modelling	*Li et al. (2022); Vićić & Harknett (2024)*
Risk	Multiple risks converging to exceed extant adaptation measures and responses	• System modelling	*Klasa et al., (2025), Goulart et al., (2024)*
Organisational Studies	Overaccumulation of non-novel changes, especially at pace, that interrupt restabilisation capacity	• Case Studies	*Girod & Whittington (2015); Rudolph et al (2002)*
Economics	Cumulative effects of early investment that overwhelm the systems normal operating parameters	• Economic modelling	*Hudson (2000)*
War studies	Combinatory leverage of multiple activities on a vulnerable node (a "victim'); regular and irregular components acting simultaneously	• Conceptual modelling<br>• Operational Research	*Huber (2002)*
